# The Enlarged Prostate from the Point of View of Practitioner and Surgeon
*A paper read at a meeting of the Society on Wednesday, 10th March, 1937.


**Published:** 1937

**Authors:** C. Ferrier Walters

**Affiliations:** Consulting Surgeon to the Bristol Royal Infirmary


### THE ENLARGED PROSTATE FROM THE POINT OF VIEW OF PRACTITIONER AND SURGEON.*

BY

C. Ferrier Walters, F.R.C.S.,
*Consulting Surgeon to the Bristol Royal Infirmary.*

---

I do not propose to enter into a long dissertation on the signs, symptoms and treatment of the enlarged prostate, but rather briefly to survey the advances in prostatectomy and the dangers and difficulties that beset the preliminary, the operative and the post-operative treatment of what may sometimes be one of the most difficult procedures in surgery to bring to a successful issue. In other words, it is of what one may call "The Prostatic Pitfalls" that I wish to speak to-night, that we may learn from the experience of others what trials and tribulations they have met with and how these have been overcome.

McGill, Belfield, Fuller and others, it is true, did a partial prostatectomy, a piecemeal removal by scissors, but it was not until about thirty-five years ago that Freyer published his first series of prostatic enucleations. From that time, lasting for nine or ten years, began what I may describe as the Dark Age of Prostatectomy. For, while Freyer obtained good results, his technique was not accurately followed; this led to a high mortality—25 per cent., and probably

* A paper read at a meeting of the Society on Wednesday, 10th March, 1937.

more—as a result of the four great bugbears: shock, hæmorrhage, sepsis and uræmia.

Freyer, by a careful selection of cases, as far as was then possible, by rapid operating, by very ample drainage, using a tube of one inch internal diameter, and by bi-daily irrigations with 1/2000 potassium permanganate solution, had lessened shock and sepsis; but hæmorrhage and uræmia still took a toll. His followers failed particularly because of inadequate drainage, which I not infrequently saw; but both he and others failed, at that time, to recognize the vast importance, the absolute necessity of re-establishing an adequate renal function before the operative procedure was attempted. A fairly healthy appearance and a clean tongue and urine may be very deceptive: only very advanced cases show all the signs of renal bankruptcy—thirst, creamy tongue, early morning nausea or vomiting and urinous breath.

I still have a vivid recollection of that massive, bluff Irishman, with a powerful pair of forearms and hands, one forefinger in the bladder, the other in the rectum, rapidly producing the prostate as a conjurer produces a rabbit from a hat. A powerful forefinger is a great asset, and there are few surgeons who have not experienced the effort sometimes necessary to remove a prostate by the old "blind" method. A colleague of mine, in poor health, found it physically impossible to remove one, and handed his cases to me.

The next great advance in prostatectomy was the two-stage operation designed to relieve back pressure on the kidneys and to improve renal function. It was, if I remember rightly, suggested by Cabot, the American urologist, in 1908, and it was found that in addition to a considerable diminution of the mortality due to uræmia it unexpectedly also reduced that due to hæmorrhage.

Every surgeon who has examined a large adenomatous prostate, at the first stage operation, will agree that often, after a week or ten days' drainage, the shrinkage of that organ is remarkable. For my part I have sometimes wondered what on earth had become of the prostate, which would be a quarter, or even less, of its original size. Most of the enlargement was obviously due to congestion, the relief of which meant less hæmorrhage at the time of enucleation. Thus another of the dangers was considerably lessened.

It was noticed at St. Peter's Hospital, at about the time I went there to see Freyer operate, that a certain number of men with enlarged prostates with acute retention—a *first* retention, that is, in, as they there described it, "a man of blameless life," meaning that he had taken very little interest in his genito-urinary apparatus—that some of these cases of retention, with the bladder up to the umbilicus, after the bladder was completely emptied by catheter, died on the seventh to ninth day. Nothing abnormal was noticed for five or six days, except contracted pupils, muscular twitchings, and a diminution in urinary output, to be followed by sudden coma and death, obviously due to uræmia. The post-mortem findings were distended bladder, ureters and kidneys. This led to an order being given to all house surgeons that in every first retention with distended bladder the bladder was to be emptied slowly through a period of forty-eight hours, either by repeated catheterizations, increasing the amount of urine drawn off each time, or by tying in a No. 12 catheter with a spigot, which was removed as necessary, or by tying in a very small

No. 1 catheter, the urine running away a little faster than it was secreted.

This procedure is now known as "gradual decompression" of the kidneys, a principle now applied to many surgical procedures, *e.g.* the slow emptying of abdominal ascites to prevent shock, of massive empyemata to prevent "mediastinal flap," of the gall-bladder, of the cerebro-spinal fluid; in hæmatocolpos, in hydramnios, and lastly in intestinal obstruction; in all these gradual decompression is of importance to avoid shock, hæmorrhage or toxæmia. The effect of this rule as to gradual decompression reduced the mortality from uræmia due to sudden bladder-emptying, save when a new, enthusiastic and uninstructed house surgeon failed to conform to it. The importance of this slow emptying is still not recognized by the profession as a whole, and I wish to emphasize how important it is if these unnecessary deaths are to be avoided.

I still get cases sent to me with the statement that "the bladder was up to the umbilicus, but that it had readily been emptied by catheter." I wonder what the outcome may be a week hence. I have seen not a few disastrous results. As previously mentioned, the post-mortem findings are a distended bladder, ureters and kidneys with atrophic cortex. The explanation of the onset of uræmia following rapid decompression of the kidneys is still *sub judice*, but it is probably due to the sudden relief of pressure causing congestion of the degenerated kidneys with resulting suppression. This does not apply, of course, to cases of repeated retention; it is the tragedy of a first retention.

As before stated, Cabot suggested a two-stage operation to improve renal function as a preliminary to prostatectomy. My first was done in 1910, and from that time to this, except on one occasion, I have always done a two-stage operation or its equivalent. The one exception was about eighteen years ago, on a man of fifty - eight, in apparently excellent condition, with an enlarged prostate, but a clean tongue and a clean urine. He died of uræmia a few days after enucleation. I say "the two-stage operation or its equivalent," by which I mean catheter drainage which I now employ in the majority of cases. But there is a certain number of cases which not only refuse to improve by this means, but actually are set back, as the result of rigors or other untoward symptoms, from the presence of a catheter: yet when suprapubic drainage is established it has the desired effect. Suprapubic drainage should always be employed in advanced cases of renal bankruptcy, where months of drainage are needed before the patients are in a fit condition to undergo the second stage. One of my cases required seven months before a normal blood-urea and other tests could be obtained.

The improvement by drainage, in my experience, is not always seen at once. The patient at first sometimes "goes down hill," with loss of flesh and appetite, a drying tongue and a lessened output or polyuria appearing. But this is usually followed by the urinary output either rising or falling to normal: the appetite returns, the tongue cleans, the general appearance obviously improves, and the tests become satisfactory after ten days' drainage. I find that a week is usually insufficient and the few extra days well spent.

The two-stage operation was slow in gaining acceptance. I remember talking to the late T. T.

Dobson, before he established the value of the blood-urea estimate, and saying that I did a two-stage operation in all cases. He said "he thought it unnecessary," and did it in 20 per cent. of cases only. I met him again a year later, and he said he then did it in 80 per cent. I think personally that it should be done in all cases, and should like to hear the opinion of other surgeons on this point.

I find that catheter drainage, where sufficient, has two advantages. One operation only is necessary, and the wound heals more readily if preliminary supra-pubic drainage has not been done, although if the wound is excised at the second operation there is little difference in the rate of healing.

At the first sign of a set-back with the first stage I anticipate further renal disturbance by continuous intravenous saline and glucose.

Drainage then lessens shock, hæmorrhage, sepsis and uræmia: but it does not eliminate any of these, and the more accurate estimation of kidney function was still to be found, though urea concentration and the dilution tests were of assistance. I have found the ratio of day to night urine of some value in advanced cases. The normal ratio of four by day to one by night being replaced by one to one is evidence of diminished kidney function, and when, as I have occasionally seen, the ratio is reversed, this is evidence of a severe grade of degeneration. One patient I had who was an old chronic syphilitic and a chronic alcoholic. He passed no urine by day, but after being in bed for about half an hour he began to pass urine freely. He died shortly after. I imagine that the explanation of this was that the kidneys were so degenerated that when, in the daytime, other tissues needed a blood supply, the kidneys' power of demand was nil; they therefore seized their opportunity at night when other organs were at rest.

I should like to hear from others their experience of the suprapubic operation for drainage only, and what dangers they have met in this apparently simple operation. For my part, under general anæsthesia, I have several times opened the peritoneum and have recognized the fact; but once I have done so without realizing it, and the result was peritonitis and death. On one occasion the small gut was wounded also; this was seen and sutured, but the patient died six weeks later of a low grade chronic peritonitis. Now I approach even this small undertaking with caution, and I always expose the bladder wall to view and partially divide it before thrusting in the de Pezzer trocar.

Drainage by catheter may be necessary in general work. Apart from its major troubles it has its minor ones, which may tend to induce the more severe. Those most frequently met with, in my experience, are blocking of the catheter by blood or mucus, or a tendency for the catheter to be partially extruded. This gives rise to retention and forcible efforts at urination. I meet the former difficulty by never using a catheter with less than two holes in it and always cutting a third hole. I have once seen a catheter with four holes: the manufacturer who was responsible for this should be encouraged rather than those who make catheters with one hole only, which is asking for trouble.

Fixation of the catheter is an important point. I have tried every type of fixation, and have returned to a simple piece of zinc oxide strapping eight or ten inches in length and tapered at one end; this end goes round the catheter, the rest round the penis, the foreskin having been pulled well forward before it is applied.

The next great addition to our armamentarium was Dobson's method of estimating the blood-urea, a more accurate, though not yet perfect, estimate of kidney function being obtained. And it is on that which I now mainly rely, together with the urea concentration and the dilution test to confirm if necessary. My first blood-urea test was done after the appearance of Dobson's paper in 1921. I now regard a return of 45 mgs. per 100 c.c. by the newer method as the normal for the old prostatic patient.

It must have occurred to all surgeons to have a patient with enlarged prostate with obviously poor renal function, and yet, before the first stage is done, to have a blood-urea returned as low as 30 mgs., yet a week later it is returned as 60 mgs. I should be glad to hear the pathologists' explanation of this. On the other hand, a blood-urea may be returned as 60 mgs., and ten days later be the same. This I regard as a worse type than that returned first as 100 mgs. and later as 60 mgs., as it shows little kidney resilience and indicates a bad risk. Both must be drained until normal, but the non-resilient type will probably need the longer drainage before normal is reached.

It is well to recognize the fact that a blood-urea of over 300 mgs. need not preclude a final successful outcome to prostatectomy. In my own experience a patient in coma with a blood-urea of 250 mgs. was successfully dealt with after four months' drainage. In another robust - looking if somewhat bucolic patient a return of 315 mgs. was made. He took seven months' drainage before normal was reached and a successful issue obtained.

Before Dobson introduced his blood-urea test, in 1919, Hugh Young, of Baltimore, produced "220" of mercurochrome This was practically a specific for bacillus coli. After proving the value of this drug in various septic conditions I applied it to prostatectomy, and since then the terrors of sepsis have been reduced to a minimum ; a rise in temperature no longer fills me with apprehension nor does a rigor terrify. The vast majority of prostatectomized patients proceed on the even tenor of the old man's temperature between 97 and 98 degrees throughout the operative period. It is used through both first and second stages, daily or every other day, with very heartening results. I regard this drug as having practically eliminated the fear of sepsis. Whether it will be replaced by ammonium mandelate remains to be seen.

Prostatectomy has passed through various stages. At one time "catheter life"—often a short one at that and by no means merry—or suprapubic drainage were the only means of overcoming permanent retention, although I found, in the past, on a few occasions in inoperable cases, that the passage of increasingly larger Lister's sounds at several sittings, left in the urethra on each occasion for fifteen or twenty minutes, enabled micturition to be re-established, the patient sometimes after many months dying of intercurrent disease, without further retention. In the same way mercurochrome by catheter or intravenously has so diminished bladder infection as to reduce bladder congestion sufficiently to clear the way for micturition again.

As I have said, a piecemeal prostatectomy was the forerunner of enucleation, but Bottine's electro-cautery was also the forerunner of prostatic resection.

This method I used on one or two occasions in the dim past. The instrument was like a lithotrite, the male blade being heated electrically. Both blades were hooked over the prostate, the current was turned on, and the male blade was slowly withdrawn, burning through the gland. The method was too primitive to stand the test of time.

Next Freyer's blind total enucleation, on which most modern treatment is based, came into vogue. Thomson Walker's open operation improved on this, but prolongation of the operation tended to increase shock. Harris's closed operation and prostatic resection are further developments. Perineal prostatectomy, which I have done on a few occasions, is still largely used in America, although even there the suprapubic route is gaining ground. With even these modern methods it is still the preparation of the patient which in my opinion is the most important factoı in success. Neglect this and with any method the mortality will soar. Most surgeons will agree that spinal anæsthesia, with its relaxation, has greatly facilitated the ease and speed of removal of the prostate.

For my part I now use a modification of Harris's operation, in that I attempt to close the bladder on the fourth day after the prostatectomy. I have given up suturing the trigone to the urethra or prostate bed because I very much doubt if the stitch holds for more than forty-eight hours, although I understand it is said to have been seen by cystoscope still intact at the end of fifteen days. So-called complete closure is a misnomer: in my experience the silkworm-gut suspending the catheter acts as a drain into the closed subcutaneous tissues and tends to cause suppuration even in the presence of mercurochrome. I find that

No. 5 or 6 catgut tends to soften in the presence of urine in forty-eight hours. In my opinion a flap of bladder mucous membrane kept in position by a gauze pack is the best protection against hæmorrhage.

In enucleating the prostate I find it best to go first through the anterior commissure which makes a tear in the mucous membrane about one-third down the vertical depth of the prostate. Then all tags having been removed under direct vision, and the prostatic capsule having shrunk to a third or less of its original size, I pack the cavity with gauze, taking care to see that the flap of mucous membrane is turned in all round on to the prostatic bed and pressed close to the walls by the gauze. In fact, I have reverted to a simpler form of operation. There has been no serious hæmorrhage in cases that I have handled entirely myself for twelve years. The last accident arose when pulling out the pack: a violent hæmorrhage occurred, leading eventually to the death of the patient. From this I learned a lesson never to pull the pack out by a straight pull, but always to twist and pull and twist and pull until it falls out. Having completed the packing, a small tube, half an inch in diameter, is introduced behind the gauze pack, the bladder is closed with a double suture up to the tube, and a stout No. 5 or 6 catgut suture or, better still, a thin kangaroo tendon is placed through the bladder wall round the tube and a half-knot only made in it. The bladder is then mercurochromed and the wound closed.

Intravenous glucose is always given after operation. The pack is left for four days, mercurochrome being run down the tube by catheter and funnel daily. This prevents sepsis, and a four-day pack practically precludes anything more than a slight ooze on its removal. This is always done under gas, the tube being removed at the same time; a No. 14 bicoudé gum-elastic catheter is then passed. The catheter is mounted on a No. 1–2–3 Lister's sound to facilitate its passage, and shortened so that the sound reaches just beyond the most distal hole in the catheter. The bladder is then thoroughly washed free of clot. The kangaroo tendon is then tied and the bladder thus closed. Following this, to prevent any clotting in the catheter, it is gently irrigated once an hour with saline for several hours. Daily irrigations are used, followed by the introduction of 1 per cent. mercurochrome, which is kept in for an hour by spigot. The catheter is left in sometimes until the wound has healed, or if there appears to be much irritation of the urethra it is left out for a few days and again replaced.

Curiously enough I have had no trouble from a long-retained catheter, except two years ago in two cases at the same time. Each developed a periurethral penile abscess which had to be opened: each developed a penile fistula. One closed rapidly under dilatation, the other, who fought shy of dilatation, has to this day an occasional drop through the fistula on micturition.

At the first sign of a drying tongue the patient is put on continuous intravenous drip of saline and glucose, which if used with discretion certainly does sometimes appear to pull the patient out of the grave. As to orchitis, Steinach I is done in most cases, but where prolonged drainage is likely to be necessary Steinach II is preferred. Despite Steinach I, one patient developed orchitis on one side. Another surgeon tells me of a similar happening. It is well, I think, to do the Steinach operation as a preliminary to the prostatectomy, that is at the time of bladder drainage.

As to other tribulations, a massive pulmonary embolism has occurred only once in my practice. The patient on getting out of bed on the tenth day died suddenly of this tragic complication. Minor and multiple embolism I have seen on several occasions with the typical sudden pain in the chest, rise of temperature and clot expectoration on the third or fourth day. Only twice has one of the emboli produced a small empyema—both in urinary cases (one in a prostate case and one in stone in the kidney case after operation). Both yielded readily to aspiration and the injection of mercurochrome. In all abdominal operations I now attempt to prevent embolism by inducing the patient to take four or five deep breaths three times a day and encouraging movement of legs and feet in the form of exercises in bed.

The mental condition of the patient in the past not infrequently caused difficulty, but this is now seldom seen with proper preliminary preparation. It was uræmic in origin. But two years ago a patient, some three weeks after operation with suprapubic wound closed, attempted suicide by cutting his throat, and inflicted four wounds on each wrist. He eventually died refusing all food. He was definitely insane and should not have been operated on, although the mental state was thought at the time to be due to uræmia.

Prostatic resection I have done on various occasions. It is, I think, unsuited to the adenomatous enlargement but suitable for the fibrous prostate and the bar, and it sometimes gives temporary relief in carcinoma. But it needs as careful preparation and is as liable to hæmorrhage, sepsis and uræmia as any other method.

Post-operative dilatation is a necessary adjunct to any form of operation, and I practise it three-monthly on all post-operative cases, increasing the interval of dilatation until there is no contraction over a period of twelve months.

Although there are various other points that I should like to consider, time is too short. But I must at least mention the possibility of eventually treating prostatic enlargement by means of male hormone. Van Capellen, of Amsterdam, last year reported favourable results in fifty cases of enlarged prostate treated by intramuscular injections of hombroel. The testicular hormones stimulate the excretion in the urine of two hormones, hombroel and manformon. These, if balanced (probably by pituitary control), maintain the normal size of the prostate. With the male climacteric diminution in the amount of hombroel occurs, with resulting enlargement of the prostate from epithelial, connective tissue and muscular hypertrophy. Although in man there is not much obvious diminution in the size of the prostate, the administration of the hormone appears in 50 per cent. of cases to have re-established normal or improved micturition for periods up to over two years.

